# Why are older adults and individuals with underlying chronic diseases in Germany not vaccinated against flu? A population-based study

**DOI:** 10.1186/s12889-015-1970-4

**Published:** 2015-07-07

**Authors:** Birte Bödeker, Cornelius Remschmidt, Patrick Schmich, Ole Wichmann

**Affiliations:** Department for Infectious Disease Epidemiology, Immunization Unit, Robert Koch Institute, Seestraße 10, 13353 Berlin, Germany; Department of Epidemiology and Health Monitoring, Division of Health Interview Surveys and European Cooperation, Robert Koch Institute, General-Pape-Straße 62-66, 12101 Berlin, Germany

**Keywords:** Germany, Influenza, Risk group, Vaccination attitude, Vaccination knowledge

## Abstract

**Background:**

Older adults and individuals with underlying chronic diseases are at increased risk of developing influenza-related complications and are target groups for seasonal influenza vaccination in many countries. In Germany, an annual national information campaign is conducted to increase influenza vaccination uptake in the target groups. However, data are lacking on knowledge and attitudes toward influenza vaccination among older adults and those with chronic diseases. The present study aimed to (i) estimate influenza vaccination uptake for the 2012/13 and 2013/14 seasons, (ii) assess knowledge and attitudes about influenza vaccination, and (iii) identify factors associated with vaccination uptake in two risk groups.

**Methods:**

Between March and June 2014, we conducted a nationwide cross-sectional survey in adults (≥18 years) living in Germany using computer-assisted telephone interviewing. We calculated weighted vaccination coverage rates in two at-risk groups. Group 1 comprised participants aged 18–59 years with underlying chronic diseases. Group 2 comprised participants aged 60+, irrespective of underlying disease. We used univariate and multivariable logistic regression analyses to identify associations between influenza vaccination uptake and sociodemographic characteristics, and to evaluate attitudes and knowledge.

**Results:**

In total, 1,519 interviews were conducted. Seasonal influenza vaccination uptake in people with underlying chronic diseases aged 18–59 years was 24 % in 2012/2013 and 23 % in 2013/2014. In older adults, uptake was 50 % and 49 % in 2012/13 and 2013/14 respectively. There were considerable vaccination-related knowledge gaps among respondents. For example, about half of the participants who aged ≥60 years and/or suffered from underlying chronic diseases believed that influenza vaccination could cause influenza. The most commonly stated reasons for not being immunized were mistrust of the vaccination (22 %) and the perception that influenza is not dangerous (21 %). For both groups, vaccination uptake was independently associated with sex, perceived severity of influenza, perceived vaccination effectiveness, and the perceived likelihood or severity of vaccination side effects. For older adults, additional factors influencing vaccination uptake were age, underlying chronic diseases, and recent advice through physician consultation.

**Conclusions:**

Influenza vaccination coverage rates in Germany remain low. Individual perceptions regarding harms and benefits are crucial in the decision-making process. Communication strategies should focus on improving understanding and perception of personal risks arising from the disease and the vaccination.

## Background

Older adults and people with underlying chronic diseases have an increased risk of developing severe influenza and influenza-related complications [1–3]. Therefore, the World Health Organization and National Immunization Technical Advisory Groups in most industrialized countries recommend seasonal influenza vaccination for these at-risk groups [4–6].

To reduce influenza-associated morbidity and mortality, European Union (EU) member states committed to the goal of attaining vaccination coverage of at least 75 % for older age groups by 2014/15 [7]. However, thus far, only the Netherlands and the United Kingdom have achieved the 75 % threshold for older adults, whereas other European countries, including Germany, are still below the projected target [8].

To raise awareness about the importance of influenza vaccination and to increase vaccination uptake, an annual national information campaign is conducted in Germany. Data on vaccination uptake in different target groups, and about knowledge of and attitudes toward vaccination are crucial to this campaign and the progress towards the EU vaccination coverage goal. As Germany has no central immunization registry, information on influenza vaccination coverage is predominantly available from population-based cross-sectional surveys [9] and from health assurance claims data [10]. However, data on attitudes and knowledge about seasonal influenza and vaccination in specific target groups are limited.

Therefore, we conducted a nationwide cross-sectional survey in Germany to (i) estimate influenza vaccination uptake in older adults and in individuals with underlying chronic diseases for the 2012/13 and 2013/14 seasons, (ii) assess attitudes toward and knowledge about seasonal influenza and seasonal influenza vaccination and (iii) identify factors associated with vaccination uptake in these two at-risk groups.

## Methods

### Study design and population

We conducted a nationwide cross-sectional survey between March and June 2014 using computer-assisted telephone interviewing. Inclusion criteria for participation were (i) being at least 18 years of age, (ii) German-speaking, and (iii) having a mobile or landline telephone. Telephone numbers were randomly generated using Waksberg’s method [11], adapted by Gabler and Häder [12] for Germany and provided by the Leibniz Institute for the Social Sciences (GESIS) in Mannheim, Germany. People who were reached by landline were selected by last-birthday-method [13]. For the mobile phone sample, interviews were conducted with the person who answered the phone. Calls took place Mondays through Fridays from 9:30 a.m. to 2:30 p.m. and from 3:00 p.m. to 8:00 p.m., and Saturdays from 10:00 a.m. to 3:00 p.m. If the number dialed gave a busy signal or rang but was unanswered, up to 10 further attempts were made to contact the number. The management of telephone calls and call-backs was automatically regulated by the computer-assisted telephone interviewing software Voxco CC3 (10.3, Montreal, Canada). The interviews were conducted in German by trained interviewers from the Robert Koch Institute.

According to a sample size calculation, 760 subjects ≥60 years were needed to estimate a coverage of 48 % (vaccination rate in 2009/10 in this age group [14]) with a confidence interval of 95 % and an error estimate of 5 %. Response and Cooperation Rates 3 were calculated as defined by the American Association for Public Opinion Research (AAPOR) [15].

### Questionnaire

We used a structured an pre-tested questionnaire that collected data on (i) behavior, attitudes and knowledge about seasonal influenza vaccination and influenza disease, (ii) information behavior concerning influenza immunization, and (iii) sociodemographic factors. The majority of survey items had been used in previous studies conducted by the Robert Koch Institute [9, 16] but were adapted to our study sample and design. All questions were self-reported and not validated.

Influenza vaccination uptake in 2012/13 and/or 2013/14 was defined as having received a flu shot in the respective season. Additionally, information on the influenza vaccination status for each child living in the respective household of the participant was collected for the 2013/14 season. To assess influenza and vaccination-related knowledge, we asked participants to agree/disagree or state “don’t know” to specific statements. We assumed that a lack of knowledge (don’t know) reflected a lack of awareness about the importance of obtaining vaccination; therefore, don’t know was defined as an incorrect answer.

We used 10-point Likert-scales to assess perceived probability of acquiring influenza disease and probability of severe side effects following vaccination (“not likely” to “very likely”), perceived severity of the influenza disease and perceived severity of vaccination side effects (“not serious” to “very serious”) as well as perceived vaccination effectiveness (“not effective” to “very effective”).

For sociodemographic factors, we collected data on age, sex, place of residence, education level, migration background and chronic illnesses such as diabetes, stroke, cardiovascular diseases, renal failure, liver diseases, cancer, chronic neurologic disease, immune deficiency, bronchitis, or asthma. Migration background was defined as described by Schenk *et al.* based on parents’ country of birth [17]. A participant was defined as chronically ill if any of the above mentioned diseases had been diagnosed by a physician during their lifetime.

Participants who were ≥60 years of age and/or suffered from underlying chronic diseases were regarded as at-risk.

### Statistical analysis

To control for possible sampling and selection biases, the sample was weighted to match national general population parameters. The basic weighting parameters were derived from the Federal Statistical Office of Germany for 2011 [18] and included the geographical region, age, sex, and education. All results are presented as weighted data unless otherwise stated.

Descriptive statistics were applied to describe sociodemographic characteristics, vaccination coverage, reasons for not being vaccinated, information behavior, and attitude and knowledge characteristics. We used Pearson’s chi-square test to compare vaccination- and influenza-related knowledge items between vaccinated and unvaccinated at-risk participants. Additionally, we conducted stratified univariate and multivariable logistic regression analyses to determine potential associations between influenza vaccination uptake and sociodemographic characteristics, attitude and knowledge items. For this purpose we stratified the study population into two sub-groups. Group 1 consisted of people aged 18–59 years with underlying chronic diseases. Group 2 comprised people aged ≥60 years, irrespective of whether they had an underlying chronic disease. Odds ratios (OR) and 95 % confidence intervals (CI) were calculated. A *p*-value of <0.05 was considered statistically significant. Variables with a *p*-value of ≤0.1 in the univariate analysis were entered in the first step of the multivariable analyses. We then removed non-significant factors (≥0.05) from the model in a stepwise backward procedure to obtain the final model. Although they were not significant in the final model, sex, age and education variables were included *a priori* and were not removed. Additionally, an interaction term (sex#chronic disease) was included in the final model for older adults. Missing data were not replaced or imputed. Statistical analyses were performed with StataSE13 (StataCorp LP, College Station, TX, USA) using complex survey methods.

### Ethical considerations and data protection

Participants were informed about study details, including data protection and privacy issues. Verbal consent was required for participants to be included in the study. Telephone numbers were generated randomly and were deleted directly after the interview. The study was approved by the German Federal Commissioner for Data Protection and Freedom of Information. All data were collected and analyzed anonymously.

## Results

### Recruitment and sample characteristics

In total, 1,519 participants were interviewed. The Response Rate 3 was 16.2 % and the Cooperation Rate 3 was 28.7 %. An overview of the study population characteristics is presented in Table 1. Overall, 55.1 % (95 % CI 51.6–58.5) of the participants were aged ≥60 years and/or had an underlying chronic disease.

**Table 1:** Characteristics of the study sample, Germany, 2014

	Study population % (95 % CI)^a^
Sex (*n* = 1,519)	
Male	47.6 (44.2–51.0)
Female	52.5 (49.0–55.8)
Age (*n* = 1,519)	
18–39 years	31.5 (28.3–35.0)
40–59 years	33.1 (29.9–36.4)
60–69 years	16.3 (14.2–18.5)
70–79 years	14.3 (12.3–16.6)
≥80 years	4.8 (3.7–6.2)
Geographic region (*n* = 1,512)^b^	
Eastern Federal States	19.1 (16.7–21.7)
Western Federal States	80.9 (78.3–83.3)
Underlying chronic disease (*n* = 1,501)	41.1 (37.8–44.5)
<60 years and underlying chronic disease (*n* = 694)	30.0 (25.9–34.4)
≥60 years and underlying chronic disease (*n* = 807)	61.2 (56.4–65.8)
Migration background (*n* = 1,497)	16.2 (13.8–19.0)
Education level (*n* = 1,495)^c^	
Low	39.7 (36.2–43.3)
Middle	28.9 (26.0–32.1)
High	31.4 (28.6–34.3)

^a^ Weighted data (totals are not weighted)

^b^ Eastern Federal States: Mecklenburg-Vorpommern, Brandenburg, Berlin, Saxony, Saxony-Anhalt, Thuringia; Western Federal States: Schleswig-Holstein, Bremen, Hamburg, Lower Saxony, Hesse, Rhineland-Palatinate, Saarland, North Rhine-Westphalia, Bavaria, Baden-Württemberg

^c^ Low: 9 years or less of school education; Middle: at least 10 years of school education; High: university entrance diploma

### Vaccination coverage

Influenza vaccination status was available for almost all participants (2012/13: 98.6 %; 2013/14: 99.9 %). Vaccination rates for the 2012/13 and 2013/14 seasons by sex, age, underlying chronic disease and residency are presented in Table 2. Overall, more than 25 % of participants (at risk and not at risk) were vaccinated against seasonal influenza. Of those who received the flu shot in 2012/13, 70.8 % (95 % CI 64.8–76.2) were also vaccinated the following season. In 2012/13 vaccination uptake in Group 2 participants (those aged ≥60 years) was 50.0 %, and in 2013/14 uptake was 49.4 %. For those aged ≥65 years, coverage was 53.1 % in both seasons (95 % CI 47.3–58.8 and 47.4–58.8 for 2012/13 and 2013/14, respectively). Vaccination coverage increased with age and was highest in persons aged 70–79 years.

**Table 2:** Influenza vaccination uptake in 2012/13 and 2013/14

	2012/13 % (95 % CI)^a^	2013/14 % (95 % CI)^a^
Total	30.2 (27.3–33.4)	26.6 (23.8–29.6)
Sex		
Male	29.4 (25.2–34.1)	22.0 (18.5–26.0)
Female	31.0 (26.9–35.3)	30.8 (26.7–35.1)
Age		
18–39 years	15.3 (10.8–21.3)	9.9 (6.7–14.6)
40–59 years	23.3 (18.8–28.7)	18.2 (14.1–23.2)
≥60 years	50.0 (45.2–54.8)	49.4 (44.6–54.2)
60–69 years	40.6 (34.1–47.4)	39.4 (32.9–46.3)
70–79 years	58.5 (50.7–66.0)	59.2 (51.4–66.5)
≥80 years	55.7 (42.5–68.1)	53.5 (40.5–66.0)
Underlying chronic disease		
Yes	41.5 (36.5–46.7)	40.4 (35.4–45.6)
<60 years	24.1 (17.8–31.9)	22.6 (16.4–30.2)
≥60 years	56.6 (50.3–62.7)	56.3 (50.1–62.3)
No	23.0 (19.5–27.0)	17.4 (14.5–20.8)
<60 years	17.7 (13.9–22.4)	11.0 (8.2–14.6)
≥60 years	40.5 (33.2–48.2)	38.4 (31.3–46.0)
Place of residence		
Eastern Federal States	44.4 (37.5–51.5)	38.7 (32.1–45.6)
Western Federal States	27.0 (23.8–30.4)	23.5 (20.6–26.7)

^a^ Weighted data

Of participants in Group 1 (people aged 18–59 years with underlying chronic diseases), 24.1 % were vaccinated in 2012/13 and 22.6 % in 2013/14. Overall, vaccination coverage declined between 2012/13 and 2013/14. The greatest decrease was observed among people not belonging to the vaccination target group (aged 18–59 without underlying chronic diseases).

Vaccination status was available for 431 children (95.8 %), of which 15.1 % (95 % CI 11.8–18.8, crude without weighting) were vaccinated against seasonal influenza. Of all sampled children, 6.7 % (95 % CI 4.6–9.5, crude without weighting) suffered from an underlying chronic disease and of these, 24.1 % (95 % CI 10.3–43.5, crude without weighting) were vaccinated during the 2013/14 season. Participants who received a flu shot in 2013/14 were more likely to have at least one child vaccinated in their household than unvaccinated participants (44.3 % vs. 13.4 %, respectively, *p* <0.001).

### Reasons for not being immunized

In all unvaccinated participants (at risk and not at risk), the most frequently stated reasons for not having received an influenza vaccination were a perception of being at low risk for influenza disease (26.7 %, 95 % CI 23.3–30.5), not having thought about influenza vaccination yet (21.5 %, 95 % CI 18.1–25.3) and mistrust of the vaccination (18.3 %, 95 % CI 15.5–21.5). Moreover, 9.8 % (95 % CI 7.8–12.3) of unvaccinated participants opposed vaccination in general (7.2 % of all participants, 95 % CI 5.7–9.0). Among the vaccination opponents, older people rejected vaccination more frequently than younger people (participants <60 years: 7.8 % vs. participants ≥60 years: 15.9 %, *p* < 0.05).

**Fig. 1:**
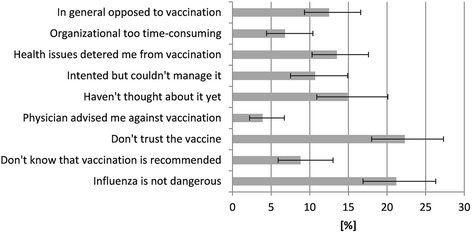
Reasons against seasonal influenza vaccination given by unvaccinated at-risk participants (*n* = 586), Germany, 2014 (weighted data; multiple answers were allowed)

Among at-risk participants, the most commonly stated reasons for not being vaccinated were (Fig. 1) mistrust of the vaccination (22.3 %), perception of low risk for influenza disease (21.2 %), and not having thought about influenza immunization yet (14.9 %). Of all participants, 5.5 % (95 % CI 3.1–9.5) of those ≥60 years and 9.7 % (95 % CI 6.1–15.1) of the chronically ill (independent of age) did not know that they belonged to a group for whom seasonal influenza vaccination is recommended.

Participants who were not at-risk felt that influenza was not a dangerous disease and considered not getting vaccinated more often than at-risk participants (31.3 % vs. 21.2 %, *p* < 0.05 and 26.0 % vs. 14.9 %, *p* < 0.05, respectively). Interestingly, at-risk participants reported mistrust of the vaccination, health issues that deterred them from vaccination and opposing vaccination in general more often than not at-risk participants (22.3 % vs. 15.5 %, *p* < 0.05, 13.5 % vs. 3.5 %, *p* < 0.001 and 12.5 % vs. 7.8 %, *p* < 0.05, respectively).

### Information seeking behavior

Of all participants (at risk and not at risk), 25.5 % (95 % CI 22.6–28.8) reported a need for further influenza vaccination-related information. Among at-risk participants, 22.1 % (95 % CI 18.5–26.1) stated an information demand. They were particularly interested in information on vaccination-related side effects (76.4 %, 95 % CI 67.3–83.6) and potential influenza-associated complications (61.4 %, 95 % CI 51.4–70.5). At-risk participants preferred to receive information from their physicians (67.7 %, 95 % CI 58.0–76.1).

### Influenza- and vaccination-related attitudes and knowledge

Among at-risk participants, the perceived probability of becoming infected with influenza was higher than the fear of vaccination side-effects (median: 5 vs. 3; both ranges: 1–10). In contrast, the perceived severity of influenza disease was identical to the perceived severity of possible vaccination side-effects (median: 5, range: 1–10 for both groups). The perceived effectiveness of seasonal influenza vaccination was higher among at-risk participants than those not at-risk (median: 6 vs. 5, both ranges: 1–10).

Differences in vaccination- and disease-related knowledge between unvaccinated and vaccinated at-risk participants are presented in Table 3. Independent of vaccination status, 53.8 % (95 % CI 49.4–58.1) of at-risk participants believed that vaccination could cause an infection. The results showed that vaccinated at-risk participants agreed that the vaccination could not cause influenza infection more often than unvaccinated at-risk participants (58.2 % vs. 38.5 %, respectively, *p* <0.001).

**Table 3:** Influenza- and influenza vaccination-related knowledge among unvaccinated (*n* = 429) and vaccinated (*n* = 586) at-risk participants, Germany 2014

	Vaccination status	Agreement % (95 % CI)^a^	*p*-value
Coughing or sneezing inside of the elbow can reduce the risk of influenza infection	Not vaccinated	68.7 (62.9–73.9)	-
	Vaccinated	72.4 (66.5–77.7)	≥0.05
Regular hand-washing with soap can reduce the risk of influenza infection	Not vaccinated	91.7 (87.7–94.5)	-
	Vaccinated	93.9 (88.7–96.8)	≥0.05
Vaccination cannot cause influenza infection	Not vaccinated	38.5 (33.1–44.3)	-
	Vaccinated	58.2 (51.5–64.6)	<0.01 **
Vaccination protects people in close surroundings	Not vaccinated	44.4 (38.7–50.2)	-
	Vaccinated	55.2 (48.5–61.8)	<0.05 *
After immunization or infection with influenza, vaccination in subsequent influenza season is necessary	Not vaccinated	74.3 (68.8–79.1)	-
	Vaccinated	79.7 (73.5–84.8)	≥0.05

^a^ Weighted data

*p <0.05; **p < 0.001

### Factors associated with vaccination uptake in individuals aged 18–59 years with underlying chronic diseases (Group 1) and older adults (Group 2)

Tables 4 and 5 present the results of the univariate and multivariable logistic regression analyses of factors influencing vaccination uptake in participants in Group 1 and Group 2 for the 2013/14 season. For both risk groups, influenza vaccination uptake was independently associated with sex, perceived severity of influenza, and perceived vaccination effectiveness. In Group 1, vaccination uptake was negatively associated with perceived severity of vaccination side effects and in Group 2 uptake was negatively associated with perceived probability of vaccination side effects. For participants in Group 2, being advised of the influenza vaccination through physician consultation in the last six months and having an underlying chronic disease were further promoters of vaccination uptake. The interaction term in the final model for older adults indicated that being female in addition to an underlying chronic disease influenced vaccination uptake, showing that women without underlying chronic disease were more often vaccinated than healthy men.

**Table 4:** Factors associated with influenza vaccination uptake in people aged 18–59 years with underlying chronic diseases, Germany, 2013/14 influenza season

	Vaccination coverage %^a, b^	Univariate OR (95 % CI)^a, b^	Multivariable OR (95 % CI)^a, c^
Sex			
Female	27.4	1.80 (0.81–4.02)	4.07 (1.50–11.03)
Male	17.3	Ref.	Ref.
Place of residence			
Eastern Federal States	33.2	2.18 (0.92–5.17)	NS
Western Federal States	18.6	Ref.	
Age			
18–39 years	18.4	NS	NS
40–59 years	24.7		
Education level			
Low	24.2	NS	NS
Middle	24.6		
High	19.1		
Vaccination cannot cause influenza infection			
Agreed	27.9	1.82 (0.82–4.08)	NS
Disagreed	17.5	Ref.	
Coughing or sneezing inside of the elbow can reduce the risk of influenza infection			
Agreed	26.0	2.18 (0.95–4.97)	NS
Disagreed	13.9	Ref.	
Perceived probability of getting infected with influenza when not immunized	−	1.28 (1.07–1.53)	NS
Perceived severity of influenza when not immunized	−	1.58 (1.18–2.13)	1.40 (1.07–1.85)
Perceived vaccination effectiveness	−	1.39 (1.19–1.62)	1.25 (1.03–1.52)
Perceived severity of side effects following vaccination	−	0.75 (0.63–0.89)	0.71 (0.57–0.88)
Perceived probability of severe side effects following vaccination	−	0.70 (0.55–0.89)	NS

Other nonsignificant variables in univariate analysis (*p* > 0.1) were: migration, being advised of the influenza vaccine through physician consultation in the last 6 months, and items focusing on influenza- and vaccine-related knowledge

^a^Weighted data; ^b^Included participants with information on relevant item; ^c^Included *n* = 166 participants with complete information on all items; *NS* not significant, *Ref*. reference category

**Table 5:** Factors associated with influenza vaccination uptake in people aged ≥60 years, Germany, 2013/14 influenza season

	Vaccination coverage %^a, b^	Univariate OR (95 % CI)^a, b^	Multivariable OR (95 % CI)^a, c^
Sex			
Female	53.4	1.43 (0.97–2.11)	--
Male	44.4	Ref.	
Underlying chronic disease			
Yes	56.3	2.07 (1.39–3.09)	--
No	38.4	Ref.	
Sex and chronic disease^e^			
Male and no chronic disease	28.2	--	Ref.
Male and chronic disease	54.1		2.10 (0.81–5.43)
Female and no chronic disease	46.6		4.80 (1.72–13.43)
Female and chronic disease	58.1		2.13 (0.73–6.19)
Place of residence			
Eastern Federal States	62.8	1.97 (1.26–3.10)	NS
Western Federal States	46.1	Ref.	
Age			
60–69 years	39.4	Ref.	Ref.
70–79 years	59.2	2.23 (1.46–3.40)	2.67 (1.42–5.03)
≥80 years	53.5	1.77 (0.97–3.20)	1.89 (0.73–4.89)
Education level			
Low	51.9	Ref.	NS
Middle	43.5	0.71 (0.47–1.08)	
High	43.8	0.72 (0.49–1.06)	
Being advised of the influenza vaccination through physician consultation in the last 6 months			
Yes	63.0	2.87 (1.89–4.37)	2.63 (1.44–4.84)
No	37.2	Ref.	Ref.
Vaccination cannot cause influenza infection			
Agreed	62.0	2.62 (1.77–3.90)	NS
Disagreed	38.4	Ref.	
Vaccination protects people in close surroundings			
Agreed	54.9	1.57 (1.06–2.33)	NS
Disagreed	43.6	Ref.	
After immunization or infection with influenza, vaccination in subsequent influenza season is necessary			
Agreed	52.5	1.64 (1.02–2.63)	NS
Disagreed	40.3	Ref.	
Perceived probability of getting infected with influenza when not immunized	−	1.44 (1.28–1.61)	NS
Perceived severity of influenza when not immunized	−	1.56 (1.41–1.71)	1.31 (1.13–1.52)
Perceived vaccination effectiveness	−	1.59 (1.45–1.74)	1.42 (1.24–1.62)
Perceived severity of side effects following vaccination	−	0.83 (0.76–0.90)	NS
Perceived probability of severe side effects following vaccination	−	0.77 (0.70–0.85)	0.69 (0.61–0.80)

Other nonsignificant variables in univariate analysis (*p* > 0.1) were: migration and items focusing on influenza- and vaccination-related knowledge

^a^Weighted data; ^b^Included participants with information on relevant item; ^c^Included *n* = 550 participants with complete information on all items; *NS* not significant; *Ref.* reference category; ^e^ *p*-value for interaction between sex*chronic disease: 0.012

## Discussion

Our study aimed to estimate seasonal influenza vaccination uptake in people aged ≥60 years and in people with underlying chronic diseases in Germany. Furthermore, influenza-related attitudes and knowledge, as well as factors influencing vaccination decision-making were analyzed. Our results revealed that vaccination coverage remained suboptimal for at-risk groups in Germany: Only 49 % of older adults and 23 % of chronically ill people aged 18–59 years were vaccinated in 2013/14.

Our findings on vaccination coverage rates are consistent with results from other European countries [6, 8]. Only the United Kingdom and the Netherlands have achieved the EU goal of 75 % vaccination coverage for older adults [8]. The different vaccination rates between EU countries might be explained by different communication activities supporting the vaccination recommendations, differences in vaccination systems and funding schemes, and also in different attitudes related to seasonal influenza vaccination. In England, general practitioners and other providers are encouraged to contact eligible patients in September and invite them to attend the clinic for vaccination [19]. However, because study methodologies differed (telephone survey vs. computerized immunization registries and clinical records), such differences in vaccination uptake must be interpreted with caution.

Compared to previous seasons, influenza vaccination uptake in at-risk groups decreased slightly in Germany, although this result was not statistically significant. Results of the nationwide survey *German Health Update* showed a vaccination coverage of 54 % in people aged ≥60 years in 2010/11 and 53 % in 2011/12. For people aged 18–59 years with underlying chronic diseases, vaccination uptake was 30 % in 2010/11 and 25 % in 2011/12 [20]. A decrease in influenza vaccination coverage in at-risk people has also been observed in other European countries after the 2009/10 pandemic [21–24]. A Spanish study found a decrease in vaccination coverage in older adults from 69 % in 2009/10 to 57 % in 2012/13 [25]. However, in countries such as England, no decrease in coverage was observed [26]. In our study, the strongest decrease in vaccination coverage was identified in individuals who did not belong to a vaccination target group: Vaccination uptake in healthy individuals aged 18–59 years declined from 18 % in 2012/13 to 11 % in 2013/14. Perceived low vaccination effectiveness, a perception of being at low risk for influenza disease, the fact that many people indicated that they had not yet thought about influenza vaccination, higher skepticism about vaccination after the pandemic season, and an initial influenza vaccination shortage in the 2013/14 season may have influenced their vaccination decision-making process more strongly than people who belonged to an at-risk group [27, 28, 14].

In at-risk people, the most commonly stated reasons against having received a flu shot were mistrust of the vaccination, a perception of being at low risk for influenza disease, and the fact that they had not yet thought about vaccination. These results were consistent with the findings of studies from other industrialized countries [29–35]. A Canadian study found that the most commonly reported reason to opt out of influenza vaccination in people aged ≥60 years and people with chronic medical conditions were low perceived susceptibility to influenza or low perceived severity of the infection, as well as a lack of interest, and lack of time or information [29]. Another study found that in Germany, forgetfulness was a primary barrier to influenza vaccination [30].

It is concerning that almost 10 % of all unvaccinated participants stated that they were opposed to vaccination in general (7 % of all participants). This was particularly true for older adults. In Germany, it has been estimated that 3–5 % of the population rejects immunizations in general [36] and one study found that 31 % had some prejudice against particular vaccines [37]. Results from another German study showed that overall, older adults perceived vaccinations as less important than younger people [35]. However, because our study focused on influenza vaccination, we could not rule out that some participants may have mistakenly referred the question to the influenza vaccination itself instead of vaccinations in general. Therefore, this result must be interpreted with caution.

With regard to factors associated with influenza vaccination uptake in older adults and people aged 18–59 years with chronic diseases, our results were similar for both target groups. In agreement with other studies, our analysis suggested that age and health conditions as well as perceptions relating to the disease and the vaccination were predictors of influenza vaccination status [32, 38–43].

We were not able to observe any association between influenza- und vaccination-related knowledge and vaccination uptake. A Canadian study suggested that knowledge played a smaller role in the vaccination decision-making process than beliefs concerning risk perception and vaccination effectiveness [33]. Betsch *et al.* found that the perceived risks of influenza disease and vaccination were key drivers of vaccination intention rather than cognitive risk estimation [44, 45]. Finally, a similar finding was demonstrated by Brewer *et al.* [46] who supported the assertion that risk was the main predictor of vaccination behavior.

Our analyses revealed a potential association between vaccination uptake and being advised about the influenza vaccine through consultation with a physician. However, it is interesting that this was only observed among older adults. Other studies have also showed that the physician recommendation is a crucial facilitator in the vaccination decision-making process but it remains unclear whether the patients’ age might play an addition role [47, 48, 32, 42]. The fact that some participants did not know that influenza vaccination is recommended for them and mentioned physicians as the desired source of influenza- and vaccination-related information underlines the important role of physicians in obtaining high vaccination coverage.

Our study has some potential limitations: (i) The response and cooperation rates as defined by AAPOR [15] were low at 16.2 and 28.7 %, respectively. However, because of our study design (using mobile and landline telephones and a complex weighting procedure), it can be assumed that data quality is good overall in terms of generalizability to the German population. However, we cannot rule out some degree of selection bias. (ii) Information on chronic underlying diseases and vaccination uptake were self-reported and could therefore be subject to misclassification. While a recently published study suggested an overestimation for self-reported seasonal influenza vaccination rates compared to vaccination registries [25] other studies found an adequate degree of reliability [49, 50]. Therefore, it can be assumed that vaccination coverage rates in our study population are rather overestimated. (iii) The sample was limited to the German-speaking population as the interviews were carried out in German. People who were not able to understand or speak German were therefore not represented in the study population, which might have introduced selection bias.

## Conclusion

In conclusion, our study results indicated suboptimal seasonal influenza vaccination coverage in Germany for people aged ≥60 years and those with underlying chronic diseases. Compared with previous seasons, vaccination coverage in at-risk groups remained stable, while coverage in the healthy population decreased considerably. While knowledge on disease- or vaccination-related issues did not influence vaccination decisions, perceptions of the disease severity and vaccination effectiveness as well as perceived likelihood and severity of vaccination side effects were key drivers for increased vaccination uptake. Additionally, many at-risk people had not thought about receiving a flu shot. Implementing reminder systems could therefore be helpful [51, 52]. As personal beliefs are crucial in the vaccination decision-making process, tailored communication strategies should focus on improving understanding about and perception of personal risks concerning the disease and the vaccination in the vaccination target groups.
